# Segmenting Cruise Consumers by Motivation for an Emerging Market: A Case of China

**DOI:** 10.3389/fpsyg.2021.606785

**Published:** 2021-03-11

**Authors:** Yue Jiao, Yating Hou, Yui-yip Lau

**Affiliations:** ^1^College of Business Administration, Shanghai Business School, Shanghai, China; ^2^Department of Education, Party School of Linhai Municipal Party Committee of C.P.C., Linhai, China; ^3^Division of Business and Hospitality Management, College of Professional and Continuing Education, The Hong Kong Polytechnic University, Hong Kong, Hong Kong

**Keywords:** cruise ships, cruise tourism, cruise passenger, motivation, classification of cruisers

## Abstract

After around four decades of fast growth, the cruise industry has become the most profitable and dynamic segment in the entire global leisure and tourism sector. Behind this growth is a significant shift in the profile of cruise consumers/passengers/tourists, with growth rates twice as fast as those of other types of tourists. China has become a strategic emerging market for the global cruise industry, quickly developing their cruise reception business and holding about 10% of the market share of global cruisers. In this paper, we examine and categorize various travel motivations of Chinese cruise tourists by means of a questionnaire via factor analysis, mean analysis, and K-cluster analysis. The results of the study indicate that Chinese cruise tourists are primarily encouraged to participate in cruise tourism by the motivational dimensions of family leisure/relaxation, natural and cultural exploration, bond/communication, social respect, tourism shopping, and cruise-promotion information sources. The strongest motivations for Chinese cruise tourists were found to be family leisure/relaxation and natural/cultural exploration. We identify four types of cruisers using the K-means cluster method. We find that for all cruiser demographics, leisure/relaxation is the most important motivational factor. Based on these results, we propose some specific solutions for expanding the customer pool in the Chinese cruise market.

## Introduction

The modern cruise industry emerged in the 1960s. Since the 1980s, the average annual growth rate of cruise tourist reception has reached about 8%, becoming one of the fastest growing and most economically efficient industries in the global leisure and tourism industry (Marti, 2004). In 2015, more than 23 million cruise tourists were received globally, and in 2016, this number was expected to exceed 24 million (Cruise Lines International Association [CLIA], 2016a). The cruise industry has a wide range of economic impacts, including industry links such as cruise ship manufacturing, cruise operations, cruise ship reception, and cruise ship consumption. The cruise industry covers transportation, shipping, oceans, ports, and tourism. In 2014, statistics from the Cruise Line International Association (CLIA) showed that the cruise industry had created more than 900,000 jobs and more than $39 million in wages and benefits, with a total economic output of nearly $120 billion (Cruise Lines International Association [CLIA], 2016b). The industry has become a new driving force for the development of the port economy, as well as for the transformation of coastal cities and regional economic cooperation.

In terms of regional distribution, the global cruise market is mainly located in North America, Europe, Asia, South America, and Australia, but the cruise industry is most developed in the United States, Germany, the United Kingdom, Italy, France, and Spain. In recent years, the international cruise industry began migrating from North America and Europe to the Asian Pacific and South America. From 2003 to 2013, although North America and Europe accounted for 85% of the market, the growth rate (66.5%) was much lower than that in other regions, especially Asia and South America (186.1%) (Lebrun, 2015). In the past decade, the growth rate of cruise tourism in the Asian Pacific region reached 123%. Under this context, China has developed into Asia’s largest cruise source market; in 2015, China hosted more than 980,000 homeport tourists, accounting for 47.4% of the Asian cruise market (Cruise Lines International Association [CLIA], 2016b). However, China is still in the initial stage of cruise tourism development. It has not attracted enough attention, and the market penetration rate is low (Sun and Feng, 2012; Sun et al., 2014; Sun and Ni, 2018). In 2014, the penetration rate of China’s cruise market was less than 0.05%, while the global average penetration rate was 1.4% (Wang, 2016). There is an average penetration rate of 2% or above in the following regions: United States (3.5%, cumulative rate 21%), Australia (3.4%), Singapore (3%), the United Kingdom (2.6%), and Germany (2%). In addition, China faces problems including the lack of existing cruise tourism culture (Sun and Feng, 2012; Sun et al., 2014; Sun and Ni, 2018), the shortage of passengers in the cruise market, and the low quality of cruise services. Attracting Chinese tourists to cruise tourism is key to ensuring the continued prosperity of China’s cruise industry, as is a thorough understanding of Chinese cruise tourists’ motivation.

From the existing literature, there was abundant empirical evidence regarding cruise tourism, but motivation was less researched. At present, research on cruise tourist motivation focuses mainly on areas in which the cruise industry is more developed, i.e., North America, Northern Europe, the Mediterranean, and Australia. Only a small amount of research has involved tourists from Hong Kong, SAR, and Taiwan. There has not been a systematic study conducted on the motivation of cruise tourists from mainland China. With the gradual maturity of China’s cruise port system and the improvement of its cruise service capacity, research on how to continue attracting domestic tourists to cruise tourism becomes increasingly crucial. Travel motivation is an internal driving force that urges tourists to participate in cruise travel. Understanding the travel motivation of cruise tourists and the differences between different demographics’ motivations can help cruise companies and industry organizations develop targeted marketing strategies to attract tourists and effectively improve customer satisfaction and loyalty. The current paper aims to systematically study the travel motivation of China’s cruise travelers and propose some targeted suggestions for how the cruise tourism industry can identify different types of tourist source markets and their psychological characteristics.

## Literature Review

### Studies on General Tourism Motivation

Motivation is what drives a person to perform a specific behavior. Likewise, travel motivation is the internal driving force prompting people to travel (Mayo and Jarvis, 1981; Vidon and Rickly, 2018; Egger et al., 2020). Several milestone studies in the early stages of tourism motivation scholarship have laid a solid theoretical foundation for the study in this area. Maslow (1987) put forward his famous “hierarchy of needs” by describing tourism motivation as the result of the combined effects of “social needs,” “needs of being respected,” and “self-actualization needs.” McIntosh and Goeldner, in the book *Tourism: Principles, Practices, Philosophies*, proposed four major motivations for tourism: health, culture, communication, and status/prestige (McIntosh and Goeldner, 1990). In the book *International Travel-International Trade*, Gray (1970) proposed two driving factors for tourism: fetishism (“push” factors) and roaming (“pull” factors), a new framework for research on tourism motivation (Xie, 2005). Later, Dann (1977) further clarified the specific role of push–pull motives, arguing that push factors cause travel desires, while pull factors play an important role in a tourist’s choice of destination. Crompton (1979) refined the push–pull theory and put forward seven push factors (including “escape,” “relaxation,” “self-exploration,” “prestige,” “regression,” “social interaction,” and “enhancing kinship relationship”) and two major pull factors (“novelty” and “education”). In one word, push and pull factors are associated with the individual and the travel destination, respectively (Pearce and Caltabiano, 1983).

In recent years, the study of tourism motivation has developed further (Skavronskaya et al., 2017; Wattanacharoensil and La-ornual, 2019). From domestic studies about domestic travel within mainland China, Zhou and Li (2015) examined the cross-cultural adaptation of the TCP Theory Motivation Scale in the context of mainland China and discovered that “nature,” “novelty,” and “self-actualization” are the most important factors for Chinese tourists, and “independence,” “social identity,” and “romance” were the least important motivations for travel. Mao and Song (2011) defined six motivation factors based on the logistics model, including “leisure,” “learning,” “discovery,” “status,” “relationship development,” and “excitement,” and further found that, of the six factors, “motivation for leisure vacation” and “motivation to improve learning” had a positive effect on revisiting intentions, while “motivation for exploration” and “motivation for acquisition of reputation,” had a negative impact on revisiting intentions. Wang and Qu (2013) identified six major types of travel motivation based on Gray’s pull-push theory: “knowledge,” “prestige,” “shopping and entertainment,” “relaxation,” “innovation,” and “relationship enhancement.” Zhang and Li (2009) specifically studied the tourism motivations of the elderly and found “seeking beauty” to be the most important motive, followed by “physical and mental health.” From the perspective of foreign studies, most scholars believe that “scenery,” “relaxation,” and “novelty” are influential travel incentives. Sung et al. (2016) divided tourism motivation into five categories, “landscape/knowledge,” “cost,” “relaxation/relations,” “novelty experiences,” and “sports/services.” Xu and Chan (2016) believe that, in addition to “scenery” and “relaxation” motivations, “self-improvement” and “accessibility” are also important factors. Jesus and Franco (2016) found that “new experiences,” “cultural exploration,” “leisurely vacation,” and “improving social and family relations” are the most important factors motivating tourists. Moon (2016) summed up most scholars studying Chinese tourists’ motivations for traveling internationally and found that “scenery,” “relaxation,” and “novelty” are the most influential tourism incentives.

### Research on Cruise Tourism Motivation

Cruising is a special method of travel that has unique characteristics inspiring tourists’ travel motivations. Existing studies on the motivations of cruise ship tourists have a strong regional bias, focusing mainly on tourists from regions in which the cruise tourism industry is more developed (e.g., North America, the Mediterranean, the Caribbean, Northern Europe, Singapore, and Australia). Only a small number of studies consider emerging cruise travel markets (e.g., Fan et al., 2015; Lemmetyinen et al., 2016; Sun et al., 2018; Kawasaki and Lau, 2020). Extant studies have found that cruise tourists’ travel motivations vary by region. For example, La Vina and Ford (2001) used a regression analysis to identify the motivations of cruise travel for American tourists and found that “cruise travel experience,” “travel companions,” “tourism costs,” “cruising experience,” and “adventures” are the top five motivational factors. Yoon and Uysal (2005) found that the major motivations for travelers on the Caribbean-Florida-Mississippi cruise route included “convenience,” “adventure,” “escape/relaxation,” “social communication,” and “climate.” They further observed that both push and pull factors had a positive impact on visitor satisfaction and loyalty. Teye and Paris (2010) conducted a study of cruise tourists visiting the Caribbean Sea and found that “relaxation” was an important push factor for tourists choosing cruise travel, and “multi-destination fusion” was an important motivational pull factor. They identified several other motivation factors, including “social communication,” “convenience,” “adventures,” and “climate,” etc. Andriotis and Agiomirgianakis (2010) found that “surprise” and “escape” were the main motivating factors for cruise tourists after studying the Mediterranean cruise market.

In terms of Chinese cruise tourists’ motivation, Qu and Ping (1999) studied the motivation of Hong Kong cruise tourists and found that “escape,” “social communication,” “appreciation of natural scenery,” and “culture” were the most important, while “accommodation conditions” and “catering,” were the deciding factors for tourists choosing to go on another cruise. Josiam et al. (2009) found that the most important motivations for cruise tourists in Taiwan included “adventure,” “enjoyment,” “enhancing social status,” “escape,” and “deepening familial ties.” In addition, Lu (2001) identified that the primary push motivation for Taiwanese cruise tourists included “lifelong learning,” “escape and relaxation,” “exploration and adventure,” “feeling a sense of belonging,” and “enhancing social status,” while pulling motivation included “environment and security,” “entertainment,” “appreciation of natural scenery,” “obtaining learning opportunities,” and “attraction of facilities and infrastructures,” etc. Fu et al. (2010) found that the primary push factors for Chinese tourists’ motivation included “purifying the mind,” “relaxation,” “escape,” “social communication,” “deepening of blood relationship,” and “cultural motivation,” while pull factors included “pursuit of freedom,” “appreciation of natural scenery,” and “recreation and entertainment.”

In addition, existing studies have found that cruise tourism motivation differs by demographic. In studying age/generational differences, Cartwright and Baird (1999) found that the most important motivation for tourists choosing cruise travel lies in the sense of relaxation, safety (i.e., no need to worry about being in a strange city at night), and self-fulfillment. Tourists under the age of 50, however, were found to be more motivated by “climate,” “recreation and entertainment,” and “children’s facilities,” and cruise tourists over 50 were more motivated by “environment and safety,” “enjoyment,” and “services” (Cartwright and Baird, 1999). Elliot and Choi (2011) found “escape from loneliness” to be the most important appeal of cruise tourism for young Americans, while “social communication” and “deepening family relationships” were the primary travel motivations for American cruise tourists born in the 1960s. American cruise tourists born in the 1980s, by contrast, valued “escape,” “adventures,” “creating eternal memory,” and other benefits most highly. Teye and Leclerc (2003) conducted research on cruise tourists of different races and found that the basic motivation of white Caucasians and ethnic minorities on cruise travel were similar. For example, both Caucasian Americans and American ethnic minorities were more concerned with the charm of the “cultural experience” brought about by a cruise’s multiple destinations. There is, however, a notable difference in the intensity of various motivations: the most important motivation for Caucasians, for example, were “social communication,” “cultural experience,” and “deepening family relationships,” while the most important motivations for ethnic minorities were “pursuit of freedom,” “leisure and entertainment,” and “cultural experience.”

Existing studies have also found that obtaining cruise travel information sources influence the motivational dimensions stimulating tourism (Cruise Lines International Association [CLIA], 2006; Jones, 2011). For example, travel guides, radio and television, website promotion, online reviews, official destination websites, and other people’s word of mouth recommendations may all contribute to tourists’ motivation to pursue cruise travel. Specifically, Jones’s research found that personal-based and internet-based information sources (e.g., “travel companions/spouses,” “other people’s word-of-mouth referrals,” “just want to go all the time,” “cruise company website,” and “destination website,” etc.), were most influential, while traditional media-based information sources (e.g., magazines, mail, chatrooms, and blogs) had little impact on cruise tourism (Jones, 2011). The study further found that information sources had a higher impact on first-time visitors than of returning tourists, which, to a certain extent, demonstrates the importance of widely available cruise information sources for large-scale markets. Similarly, the International Cruise Association’s research also showed “word of mouth recommendation,” “just wanting to go,” “cruise destination website,” “travel companions,” and the “cruise company website” to be the most important motivations for international cruise tourists (Cruise Lines International Association [CLIA], 2006).

Apart from differences in the classification of the motivational dimensions, the majority of existing studies put forth very similar single motivation indicators for cruise travel. It can be said that the measures of cruise travel motivation have been relatively stable and have a good reference value. For this reason, for the selection of individual motivation factors and the questionnaire design, this article first uses a frequency analysis to categorize different cruise travel motivation indicators (as shown in Table 1) according to the existing literature and select the standard measurements with the highest frequency. Based on Prebensen et al. (2013), Fan et al. (2015), Lemmetyinen et al. (2016), and Sun et al. (2018), we further develop a theoretical construct in our research study. Social recognition, relaxation, enjoyment, self-esteem, discovery, socialization, and convenience and value are the key motivational factors within the theoretical construct. As expected, it may further reinforce the tourism marketing studies.

**TABLE 1:** Summary of cruisers’ motivations.

No.	Motivation	Literature
1	To escape and relax	Cartwright and Baird (1999); Qu and Ping (1999); Lu (2001); Yoon and Uysal (2005); Cruise Lines International Association [CLIA] (2006); Josiam et al. (2009); Andriotis and Agiomirgianakis (2010); Fu et al. (2010); Teye and Paris (2010); Elliot and Choi (2011); Jones (2011)
2	Adventure	La Vina and Ford (2001); Lu (2001); Yoon and Uysal (2005); Josiam et al. (2009); Andriotis and Agiomirgianakis (2010); Teye and Paris (2010); Jones (2011)
3	Socializing	Cartwright and Baird (1999); Qu and Ping (1999); Teye and Leclerc (2003); Yoon and Uysal (2005); Fu et al. (2010); Teye and Paris (2010); Elliot and Choi (2011)
4	Leisure	Cartwright and Baird (1999); Qu and Ping (1999); Lu (2001); Teye and Leclerc (2003); Fu et al. (2010)
5	Relationship bonding	Teye and Leclerc (2003); Josiam et al. (2009); Fu et al. (2010); Elliot and Choi (2011)
6	To enjoy nature	Qu and Ping (1999); Lu (2001); Cruise Lines International Association [CLIA] (2006); Fu et al. (2010)
7	Climate	Cartwright and Baird (1999); Yoon and Uysal (2005); Teye and Paris (2010)
8	Culture	Qu and Ping (1999); Teye and Leclerc (2003); Fu et al. (2010)
9	Partner	La Vina and Ford (2001); Cruise Lines International Association [CLIA] (2006); Jones (2011)
10	To improve social states	Lu (2001); Josiam et al. (2009)
11	Convenience	Yoon and Uysal (2005); Teye and Paris (2010)
12	Fun	Cartwright and Baird (1999); Josiam et al. (2009)
13	Environment and safety	Cartwright and Baird (1999); Lu (2001)
14	Facilities and services	Cartwright and Baird (1999); Lu (2001)
15	Freedom	Teye and Leclerc (2003); Fu et al. (2010)
16	Travel cost	La Vina and Ford (2001); Cruise Lines International Association [CLIA] (2006)
17	Always wanted to go	Cruise Lines International Association [CLIA] (2006); Jones (2011)
18	Cruise website	Cruise Lines International Association [CLIA] (2006); Jones (2011)
19	Word of mouth	Cruise Lines International Association [CLIA] (2006); Jones (2011)
20	Hotel	Qu and Ping (1999)
21	Food	Qu and Ping (1999)
22	To gain a feeling of belonging	Lu (2001)
23	Rejuvenation	Fu et al. (2010)
24	Service	Cartwright and Baird (1999)
25	To create lasting memories	Elliot and Choi (2011)
26	Cruising experience	La Vina and Ford (2001)
27	Learning	Lu (2001)
28	Passenger satisfaction	La Vina and Ford (2001)

### Investigating Cruisers’ Segmentation

Market segmentation has become a useful tool in planning relevant marketing strategies (Park and Yoon, 2009). Based on Middleton (2002), segmentation is currently identified as the process of dividing a total market such as all visitors or a market sector like holiday travel, into segments or subgroups for marketing management objectives. Its objective is to enhance more cost-effective marketing under the promotion, formulation, and delivery of purpose-designed products that fulfill the identified needs of target groups. The majority of segmentation studies cope with key variables including income, occupation, family life cycle, social class, personality, and educational level (Cunningham and Crissy, 1972). Specifically, target market selection is a significant step in creating a marketing strategy. The usefulness of market segmentation in travel literature has well been identified (Jang et al., 2004). Indeed, cruises are a remarkable sector of the travel industry, market segmentation is considered as a strategic tool to obtain greater efficiency in the supply of product to fulfill identified demand, improve cost effectiveness in the marketing process (Park and Yoon, 2009), and investigate the association between cruisers and destination (Bloom, 2004).

More specifically, market segmentation in cruise research aims to encourage cruise tourism service providers to examine new cruise tourism product opportunities (Beane and Ennis, 1987). Identifying specific cruiser segments fosters the comparison of variables by groups to support management in preparing market-oriented strategies and the investigation of the distinctions between first-timers and repeaters in a less subjective approach. To a certain extent, destination management is an effective way to change their resource allocations to keep their clientele (Petrick and Sirakaya, 2004). In past literature, a number of studies have used the notion of segmentation in the cruise industry. Backman and Crompton (1991) employed a segmentation tool utilizing both behavioral and psychological measures to determine the main differences between repeaters and first-timers and evaluate the distinction between the segments’ satisfaction, perceived value, and word of mouth. Petrick (2001) conducted a research study to segment cruisers according to their perceptions of a cruise line’s reputation and recognize the differentiation between the resultant reputation groups. Later, Petrick (2005) conducted a study to segment cruisers’ purchase behavior according to their price sensitivity to find out if price-sensitive markets are desirable. Park and Yoon (2009) collected 252 tourists to segment and profile the motivations of tourists to foster an understanding of rural tourism in Korea. Similarly, Rid et al. (2014) carried out a survey of 430 tourists in the Gambia to explore market potential for rural tourism. Using a combined factor-clustering method can enhance the investigation of four distinct market segments. Under a factor-clustering approach, it recognized four main segments, namely family togetherness seeker, passive tourist, want-it-all seeker, and learning and excitement seeker. A number of approaches of tourist segmentation appeared to pertain to *a posteriori* or factor-cluster segmentation and *a priori* or criterion segmentation in the past while artificial neural networks generated a better approach in yielding market segmentation now (Mazanec, 1992; Brida et al., 2014).

Until now, socio-economic and demographic features have mainly been adopted as the starting point of segmentation. In recent years, marketers have increasingly indicated that the most effective predictor of tourist behavior should be the behavior itself (i.e., benefits and motivation) and market segmentation should evolve from behavioral theories like recreational specialization or motivation (Park and Yoon, 2009; Rid et al., 2014). Likewise, consumer behavior theory proposes motivation views as core determining factors of human behavior and consumer choice (Schiffman and Kanuk, 1978). In doing so, Loker-Murphy (1997) recognized four motivation-based clusters of backpackers in Australia (i.e., relaxers/escapers, excitement/social seekers, achievers, and self-developers). Hsu and Li (2016) developed a valid and reliable measurement instrument for creating cruise motivation in Hong Kong and mainland China. To the best of the authors’ knowledge, few academic studies have concurred in their determination to the current research work, (i.e., to examine and segment different travel motivations of cruise tourists). In this paper, we suggest some particular solutions for enlarging the customer pool in the Chinese cruise market and highlight cruise travel intention to facilitate marketers and managers in planning for the forthcoming years (Hsu and Li, 2016).

## Materials and Methods

In selecting motivational factors, the current research adopted two methods. First, initial indicators were selected based largely on their frequency of occurrence in existing literature and industry practices. Second, cruise tour leaders and experts were interviewed to conduct a pilot study. The main objective of the pilot study was to make sure the measurement indicators included all relevant dimensions of motivational factors adequately and ensured the statement of survey instruments was clear and comprehensively worded to gather all relevant data in our study. Hence, we invited a group of cruise tour leaders and experts to examine the content (Rao et al., 1999). In the content validation process, we validated the motivational factors through increasing, decreasing, refining, and finalizing the list of motivational factors included. In particular, confirmation of the match between measured dimensions and Chinese tourists, among which interviewees and the researchers mainly included experts from research institutions (6 persons), cruise tourists recruited via the travel agency (20 persons), and cruise tour leaders of a charter travel agency (10 persons). Given the characteristics of outbound Chinese tourists, motivational dimensions such as “gaining face” and “shopping” (duty-free goods, etc.) have been included in the scale.

Since China’s cruise tourism industry started relatively late, there are still problems such as insufficient publicity and promotion, a lack of cruise travel culture, low product awareness, and low market penetration (Sun and Feng, 2012; Sun et al., 2014; Sun and Ni, 2018). At present, Chinese tourists have limited understanding of cruise products. Studies have demonstrated that specific information sources can effectively trigger cruise tourists to travel (Whyte, 2017; Sun et al., 2019a, b). For example, before exposure to cruise information, Chinese tourists were relatively unfamiliar with cruise products, and their intent to travel was often not strong. After obtaining cruise knowledge from various media, websites, and verbal information sources, however, we expect that cruise travel motivations would be easily triggered. From the industry practice perspective, international cruise associations also consider information sources as an important motivating factor. Thus, both in theory and practice, there is reason to include information sources into a motivational scale for cruise tourism. Therefore, this article has included information sources in its motivational scale. The final questionnaire included two sections: (1) the motivational measurement section, and (2) the tourist demographic information section, in which the motivation measurement section contained 37 items which were shown in the previous section. The items were measured using a 5-point Likert-type scale ranging from 1 (strongly disagree) to 5 (strongly agree).

Because the current Chinese cruise market adopts the “ship-chartered mode” for cruise ticket sales, this questionnaire was distributed and administered via the “Shanghai Bus International Travel Co., Ltd.” The target vessel for this survey was the Mariner of the Seas from Royal Caribbean International, a ship traveling via Japan and South Korea (the “Shanghai-Osaka-Beppu-Shanghai” seven days six nights tour) with the Shanghai Wusongkou International Cruise Port as the home port. Regarding the sample size, the sample size was expected to be more than five times that of the survey items. In our questionnaire, we set 37 items. Thus, we determined to collect at least 185 questionnaires (Tinsley and Tinsley, 1987; Comrey, 1988). As such, the chartered-cruise consisted of 90 tour groups with about 40 people per group. In the study, the survey questionnaires were distributed and invigilated by the cruise tour leader(s). During the survey administration process, the cruise tour leader(s) briefed the tourists and controlled the progress of completion. Finally, 360 questionnaires were distributed to nine tour groups, and a Korean beauty soap was offered as a token of appreciation for participating tourists. Finally, 325 copies of the completed questionnaires were returned, with a response rate of 90.3%. A *t*-test was conducted for the missing values, the level of significance was at 0.03 (*p* < 0.05), indicating that the missing values were not completely missing at random, therefore, the linear trend method was used to fill in missing values.

In terms of data processing, the current study used SPSS 22.0 for statistical analysis. To identify cruise travel motivations, the current study adopted a factor analysis to reduce the dimensions of the 37 motivational factors and extracted six major travel motivational dimensions for cruise tourists in mainland China, including “social respect,” “information source,” “leisure/relaxation,” “natural and cultural exploration,” “bond/communication,” and “shopping.” For tourist classification, the current paper used K-means cluster analysis to classify four types of cruise tourists based on the six motivational dimensions obtained from the factor analysis. A specific discussion of the analysis results is presented below.

## Results

### Demographics of Participants

The demographic characteristics of respondents are summarized in Table 2. A total of 48.6% of the cruise tourists surveyed were men, and 51.9% were women. Nearly 50% of tourists were over 45 years old, with the majority of tourists being middle-aged or elderly tourists. In terms of educational level, more than 60% of the tourists had received higher education, and, of these, 31.8% had received vocational or college education, and 33% of tourists had a bachelor’s degree or above. In terms of job and occupation; retired people accounted for the majority of tourists surveyed (43.1%). Close to 50% of the tourists had an annual household income between 50,000 RMB and 150,000 RMB, and the vast majority (87.6%) had an annual household income of less than 300,000 RMB, indicating that most cruise tourists held mid-level incomes. In terms of travel experience, the majority (78.8%) of tourists did not have experiences of traveling abroad. Seventy percent of the tourists chose to travel with their families. More than 80% of tourists indicated that they would choose Shanghai as their departure city for future travels, indicating that Shanghai would be a potential city to develop as a cruise home port in the future.

**TABLE 2:** Demographic characteristics of respondents.

Variable		Proportion	Variable		Proportion
Gender	Male	48.6%	Annual income	0–4.99	7.8%
	Female	51.4%		5–9.99	21.8%
Age	<18	0.6%		10–14.99	25.1%
	18–24	6.1%		15–19.99	24%
	25–30	13.4%		20–29.99	8.9%
	31–35	14.5%		30–39.99	3.9%
	36–45	11.2%		40–49.99	1.7%
	46–55	9.5%		>50	6.7%
	56–64	22.9%	Oversea experience	No	78.8%
	>65	21.8%		Yes	21.2%
Education level	<High school	13.4%	Work status	Full time	36.1%
	High school	21.8%		Part time/self-employed	10.7%
	Vocational college	31.8%		Retired	43.1%
	Undergraduate	30.2%		Student	3.2%
	Graduate	2.8%		Other	6.9%

Based on the above analysis, it is evident that the Chinese cruise tourists surveyed were largely middle-aged and elderly people with mid-level incomes. These demographic groups have more leisure time and disposable income. Though middle-aged and older people still make up much of the current Chinese cruise market, the cruise market in China tends to be younger (Lau et al., 2014). For this reason, the design of China’s cruise tourism products should effectively promote the travel motivations of younger tourist groups.

### An Exploratory Factor Analysis of Cruise Travel Motivation

#### Results of Factor Analysis

Prior to the analysis of the 37 motivational items, the data were tested for feasibility using the KMO statistical test and the Barlett’s Test of Sphericity. The results found a KMO level of 0.859, which is greater than 0.7 and the Barlett sphere test value was 4065.967, with a significant value of 0.000, indicating that the correlation coefficient matrix of the variables was a non-unit matrix, and the data were suitable for factor analysis.

The current paper adopted principal component analysis to extract common factors and then rotated the extracted common factors through the maximum variance rotation method to obtain a rotation component matrix. The results of the exploratory factor analysis are shown in Table 3. Six common factors were extracted with a cumulative variance contribution rate of 61.483, indicating that these common factors can better explain the original measures. In addition, the factor loading for all motivational items were found to be greater than 0.4, and thus the 37 motivational items can be categorized into six dimensions for follow-up studies. Furthermore, by calculating the internal consistency coefficient (Cronbach’s Alpha value), the overall Cronbach alpha for the scale was found to be 0.931, and the 6-dimension coefficients were all greater than 0.7, indicating that the scale had high reliability.

**TABLE 3:** Exploratory factor analysis of Chinese cruisers’ motivations.

Motivation dimension	Loading						Accumulative rate (%)	Cronbach’s α
	X_1_	X_2_	X_3_	X_4_	X_5_	X_6_		
**Factor 1: social respect**							16.152	0.922
To gain a feeling of belonging	0.836							
To increase self-worth	0.820							
To help me feel like a better person	0.814							
To gain status/face	0.798							
To derive a sense of accomplishment	0.778							
To challenge my abilities	0.687							
Low travel cost	0.632							
**Factor 2: information source**							14.636	0.888
Internet advertisement		0.793						
Travel blog		0.733						
Online retailers		0.722						
Social media		0.720						
Television/radio commercial		0.718						
Newspaper/travel magazine		0.680						
Cruise company website		0.609						
Destination website		0.589						
Spouse/partner/travel companion		0.557						
Always wanted to go		0.556						
Word of mouth		0.531						
Travel agent recommendation		0.461						
**Factor 3: leisure/relaxation**							10.948	0.833
To mentally relax			0.786					
To physically relax			0.762					
To have fun/be entertained			0.724					
To interact with family			0.679					
To escape from the hustle of daily life			0.595					
To seek equilibrium			0.585					
To taste local cuisine			0.535					
**Factor 4: natural and cultural exploration**							9.430	0.858
To discover new places				0.792				
To visit sites				0.773				
To experience difference cultures and lifestyle				0.747				
To enjoy nature and scenery				0.728				
To experience pleasant weather				0.619				
**Factor 5: bond/communication**							5.824	0.824
To build friendships with others					0.627			
To take adventure/seek novelty					0.592			
To enhance friendship					0.559			
To enrich myself intellectually					0.533			
**Factor 6: shopping**							4.492	0.700
To buy local crafts						0.559		
To buy general or duty-free products						0.421		

In order to obtain the aggregate validity and discriminant validity of the measurement scale, the confirmatory factor analysis was performed in AMOS 17.0. The measurement items were significant at the 0.001 level (*P* < 0.001). Also, all composite reliability (CR) values were greater than the minimum threshold of 0.700, ranging from 0.702 to 0.924. It indicated that the internal consistency among measurement items for each factor was good (Hair et al., 2010). To examine the discriminant validity, the average variance extracted values were calculated and Pearson analysis was performed. As shown in Table 4, all AVE values exceeded the suggested cut off of 0.500, and the square root of AVE was larger than the correlation coefficient between each pair of constructs. It showed that the discriminant validity of the scale was satisfactory (Fornell and Larcker, 1981).

**TABLE 4:** Discriminant validity.

	Social respect	Information source	Leisure/relaxation	Natural and cultural exploration	Bond/communication	Shopping	AVE (CR)
Social respect	**0.798**						0.636 (0.924)
Information source	0.379	**0.638**					0.508 (0.889)
Leisure/relaxation	0.331	0.333	**0.640**				0.512 (0.828)
Natural and cultural exploration	0.437	0.288	0.462	**0.744**			0.554 (0.860)
Bond/communication	0.646	0.322	0.490	0.480	**0.746**		0.557 (0.832)
Shopping	0.540	0.349	0.390	0.269	0.531	**0.737**	0.543 (0.702)

*Diagonal elements in bold are square roots of AVE. Off-diagonal elements are the correlations between constructs.*

#### Cruise Travel Motivation Dimension Identifiers

According to the factor loading matrix and the meaning of the items included in each common factor, and further referring to the existing literature’s conclusions on the dimensions of cruise travel motivation, the six motivational dimensions extracted can be renamed.

Dimension 1 is named “social respect” because the items “to gain a feeling of belonging,” “to increase self-worth,” “to help me feel like a better person,” “to gain status/face,” and “to derive a sense of accomplishment” are closely related to different ways of achieving social respect. The mean values of all indicators were above 3, with “to increase self-worth” having the highest mean value of 3.27, followed by “to derive a sense of accomplishment” (3.23); “to challenge my abilities” (3.2); and “to gain status/face” (3.01), indicating that Chinese tourists potentially use cruise tourism to satisfy their sense of belonging, achievement, and pride. This may be because China’s cruise tourism industry is at an early stage, and the cultural awareness of cruise products is low, so, influenced by the traditional concept of the luxury cruise, people often regard cruise tourism as high-end tourism and believe that participating in cruise tourism is a symbol of status.

Dimension 2 is named “information source.” Promotion information is a non-negligible factor stimulating the tourism motivations of potential cruise tourists. Among them, other people’s “word-of-mouth” (mean value of 3.5) had the greatest impact on tourists opting for cruise travel. In addition, “travel agent recommendation,” “cruise company website,” “social media,” and “internet advertisement” also had a great impact on tourists. Currently, the Chinese cruise market mainly adopts either charter ships or block group space for ticket sales, so tourism agency promotion can effectively stimulate tourists’ travel motivation. In addition, the impact of internet information such as official websites, social platforms, online games, and online reviews on cruise tourists should be further expanded.

Dimension 3 is titled “leisure/relaxation,” and it mainly embodies tourists’ motivation for relaxation, enjoying fun, and promoting family harmony. Among them, “to mentally relax” and “to physically relax” reached means of 4.15, ranking first out of the 37 motivational indicators, followed by “to have fun/be entertained” (3.94). From the tourists’ perspective, the motivational index scores under this dimension were higher, indicating that the motivation for cruise travel for most Chinese tourists was the fulfillment of “relaxation/escape” and “joyfulness” through family travel. Thus, international cruise ships are equipped with abundant on-board entertainment facilities and various entertainment activities, thereby creating a relaxing, entertaining, and family friendly atmosphere for family leisure.

Dimension 4 is named “natural and cultural exploration,” as it includes motivations to explore the destination’s nature, enjoy its pleasant climate, and experience its unique culture, all of which reflects the “adventurous” mentality of tourists. Among these motivations, the score of “to discover new places” was the highest (3.89), indicating that the multi-destination characteristics of cruise travel have a significant stimulating effect on tourists’ motivation to travel. It can be said that the “multi-destination integration” allows tourists to experience a variety of unique cultures and lifestyles as they travel through different regions. Therefore, this advantage should be highlighted during promotion and publicity.

Dimension 5 is titled “bond/communication” and mainly reflects tourists’ desires to make friends and gain knowledge. In this dimension, “to enrich myself intellectually” had the highest mean (3.51), followed by “to enhance friendship” (3.45) and “to build friendships with others” (3.22). From a social perspective, tourists choose cruise tourism first for the needs of gaining knowledge, followed by the promotion of friendship with friends and the possibility of making new friends through social activities onboard or onshore. Thus, it can be concluded that cruise travel companies should appeal to tourists’ curiosity by, for example, opening up more public spaces on cruise ships, increasing on-board training, or planning activities that promote interaction between cruise travelers.

Dimension 6 is named “shopping” and includes the motivations “to buy local crafts” and “to buy general or duty-free products.” Cruise tourism itself is an outbound tourism activity, and Chinese tourists have distinctive shopping-orientation characteristics in terms of outbound travel. Therefore, the deployment and promotion of duty-free shops on cruise ships could significantly stimulate demand for cruise travel among Chinese tourists. Furthermore, during cruises, tourists can visit and purchase items at multiple destinations. When designing and planning tourism products, companies should incorporate more regional and cultural products and create unique regionally imprinted souvenirs (Volo, 2021).

#### Differences in the Motivational Dimensions of Cruise Consumers

**FIGURE 1:**
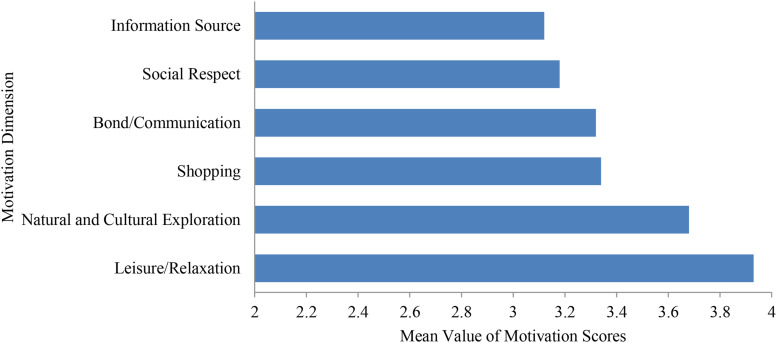
Comparison of cruise motivation dimensions.

By comparing the motivational dimensions of all tourists (as shown in Figure 1), it was found that “leisure/relaxation” and “natural and cultural exploration” were the main motivating factors, which is consistent with the findings of most international cruise passengers’ motivation studies. In addition, for Chinese cruise passengers, travel shopping was an important motivating factor, as it is for current domestic outbound shopping characteristics. Social motivation also had a great impact on the Chinese cruise passengers’ outbound travel activities. Since cruise tourism is still at its early stages in China, there is a lack of cruise culture, a low awareness of cruise products, and limited access to information on cruise tourism, which explains the current study’s finding that information sources have a lesser impact on cruise passengers.

In terms of demographics, a significant test of gender difference and tourist motivation found that, apart from “natural and cultural exploration,” the difference in cruise travel motivation was not significant (*p* > 0.05). The results indicated that, compared to male tourists, female tourists were more motivated by the natural and cultural exploration experiences brought on by cruise tourism. Therefore, the cruise industry can more effectively target the female market by promoting the experiences of nature and culture provided by cruise routes and destinations. In terms of the number of cruise trips, analysis of homogeneity of variance was first performed before conducting one-way ANOVA. Factors with a significance level greater than 0.05 were corrected using the LSD method. The remaining factors were corrected using the Games-Howell method. Afterward, the LSD test found that, apart from the significant difference in “information source,” there was no significant difference observed in the other motivation dimensions, indicating that the experience of tourists participating in cruise travel had an impact on the information source. Research shows that the mean value of “information source” was highest for revisiting tourists. This is likely because, for tourists with experience in cruise travel, the influence of information sources, especially online information has been increasing. This demonstrates the importance of publicity and promotion. In addition to the above findings, there were no significant differences in other demographic information of Chinese cruise passengers’ travel motivations. Therefore, simply identifying tourists’ demographic differences cannot necessarily promote an in-depth understanding of tourists’ travel motivations. The industry should further explore and identify different types of cruise passengers based, not on demographic features, but on the motivational dimension. This would help companies conduct effective market categorization and create targeted marketing strategies motivating different customer groups to travel.

### Classification of Cruise Passengers

#### K-Means Clustering Analysis

**FIGURE 2:**
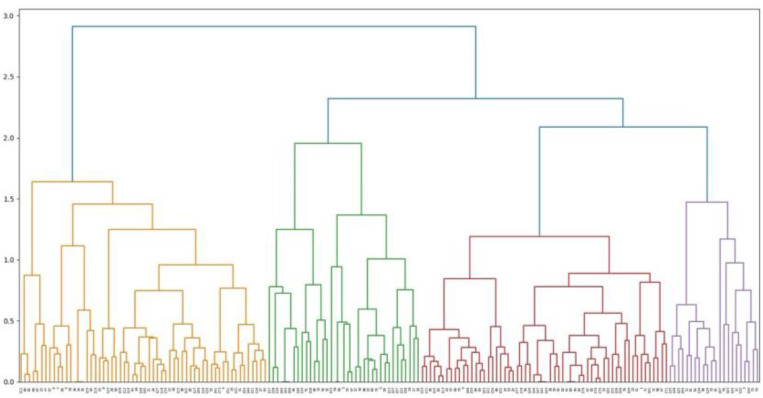
Visualized hierarchical clustering.

**FIGURE 3:**
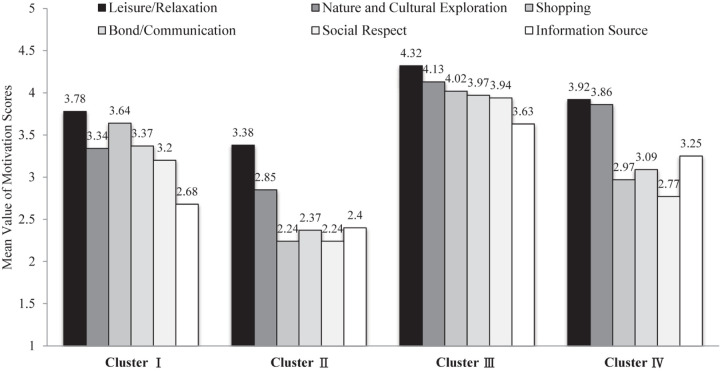
Cluster centers of motivation dimensions for each group.

This paper used K-means clustering to classify tourists based on the six major dimensions of cruise travel motivation. K-means clustering classifies tourists based on the cluster means. For a category, the score of all tourists in this category was closer to the cluster mean of that category as it was to that of the cluster means of other categories. It can be said that the cluster mean of the final iteration of K-means clustering reflects the category mean to some extent. In order to determine the value of K, the hierarchical clustering analysis was performed by the Python software. As shown in Figure 2, cruise tourists were classified into four main categories. Therefore, the K value was selected as 4. In the end, cruise passengers were classified into four categories, as shown in Table 5. Scheffe’s *post hoc* test shows that no significant differences were detected for “information channel” on Type I and Type II tourists and “leisure/relaxation” on Type I and Type IV (*p* > 0.05), but significant differences were detected for the other 34 pairs of comparisons (*p* < 0.05), indicating that the clustering results were reasonable. Figure 3 shows the different clustering centers of different motivational dimensions for the different tourist categories.

**TABLE 5:** The cluster results of cruisers.

Tourist clusters Motivation dimensions	Cluster 1	Cluster 2	Cluster 3	Cluster 4	F-statistics	Sig	Scheffe’s *post hoc* testing between binary clusters (Sig)					
							1–2	1–3	1–4	2–3	2–4	3–4
Social respect	3.20	2.24	3.94	2.77	65.556	0.00	0.00	0.00	0.05	0.00	0.02	0.00
Information source	2.68	2.40	3.63	3.25	45.645	0.00	0.23	0.00	0.00	0.00	0.00	0.02
Leisure/relaxation	3.78	3.38	4.32	3.92	27.225	0.00	0.01	0.00	0.52	0.00	0.00	0.00
Natural and cultural exploration	3.34	2.85	4.13	3.86	38.280	0.00	0.01	0.00	0.00	0.00	0.00	0.01
Bond/communication	3.37	2.37	3.97	3.09	62.946	0.00	0.00	0.00	0.05	0.00	0.00	0.00
Shopping	3.64	2.24	4.02	2.97	75.122	0.00	0.00	0.02	0.00	0.00	0.00	0.00

In order to further verify the rationality of the K-means clustering analysis, discriminant analysis was conducted. Results are shown in Tables 6, 7. The three discriminant functions explained 100% of the variables, indicating that clustering had significant differences and discriminative power. For each cluster, the values of precision, recall, and F1-score exceeded 70%. This shows that the credibility of clustering was good.

**TABLE 6:** The Eigenvalues.

Function	Eigenvalue	Percentage of variance	Accumulation	Canonical correlation
1	1.496	38.7	38.7	0.774
2	1.356	35.1	73.7	0.759
3	1.017	26.3	100	0.710

**TABLE 7:** Training set prediction accuracy.

Category	Precision	Recall	F1-score
Cluster 1	87.06%	100.00%	93.08%
Cluster 2	94.74%	91.53%	93.10%
Cluster 3	100.00%	71.67%	83.50%
Cluster 4	100.00%	72.23%	83.88%

##### Type I: Psychocentric tourist

The age of these tourists was relatively older, in which more than 30% of the tourists were over the age of 65 and the average values of their six common motivations were all in the middle level. Among them, “leisure/relaxation” had the highest mean score, followed by “shopping.” “Information source” was the weakest with a mean score of 2.68. These results suggest that this type of tourist participates in cruise travel mainly to meet the family’s needs of entertainment and travel shopping, while information sources (website advertisements, online platforms, and other people’s word-of-mouth recommendation, etc.) have little impact on their travels. From the perspective of the subdivision motivation index, the values of “to mentally relax,” “to physically relax,” and “to have fun/be entertained” scored the highest in terms of “leisure/relaxation.” It can be inferred that these tourists are not sensitive to information provided by the internet and others, nor are they good at actively obtaining cruise information, but they prefer a calm atmosphere and familiar entertainment activities and they have the desire to travel to multiple destinations abroad. They will see cruise tourism as a form of traditional outbound travel. According to Plog’s psychological type model, this type of tourist possesses the psychological characteristics of being psychocentric and mid-centric, and their travel motivations are likely triggered by leisure holiday products and travel shopping.

##### Type II: Traditional tourist

The average scores of the six major motivations for this category of tourist were all at the lowest level, indicating that the motivations for this type of tourists were not obvious in all aspects. Among them, “leisure/recreation” had a higher score, indicating that this type of tourist may choose cruise travel mainly to enhance family relationships and entertainment whereas motivations such as social respect and shopping had little influence. From the subcategory indicators, the scores for “to mentally relax,” “to physically relax,” and “to interact with family” under “leisure/relaxation” were highest. These tourists belong to the “dogs” market of the BCG matrix, and their motivations are not obvious and are, thus, difficult to motivate. According to Plog’s psychological type model, this type of tourists’ psychological characteristics are obviously psychocentric, and they are conservative, less adventurous, more willing to stay with their family, prefer routines, and reject new things. The product promotion strategy toward such tourists should combine the characteristics of cruise tourism, outbound travel, and family leisure vacations through traditional channels.

##### Type III: Pioneer

The level of education of this category of tourists is relatively high. Nearly 40% of them have a Bachelor’s degree, and the scores of the six major motivation factors were all higher (more than 3.6) than for other types of tourists, especially for “leisure/relaxation” and “natural and cultural exploration.” In terms of subcategory indicators, the scores of “to mentally relax,” “to physically relax,” and “to have fun/be entertained” under “leisure/relaxation” were the highest. These tourists incline toward leisure, entertainment, and multi-destination exploration; they tend to like adventure and exploration, and they are good at accepting new things, are very communicative and active, and possess the all centric characteristics of Plog’s psychological types. As an emerging form of tourism, cruise tourism can meet tourists’ desires for taking adventure, seeking novelty, and experiencing difference cultures and lifestyle. In the emerging cruise market, companies can attract highly educated and younger potential customers through a full range of cruise culture communication channels.

##### Type IV: Sightseer

The mean values of the six motivation factors of this type of tourist are all in the middle level. Among them, “leisure/relaxation” and “natural and cultural exploration” had a greater impact on cruise travel behavior, while “social respect” had the weakest influence. From the perspective of individual motivation indicators, the experience of different cultures and lifestyles, relaxation, enjoyment, and escape are this type of tourist’s main motivating factors. These tourists pay attention to the core functions of cruise tourism (i.e., family leisure and entertainment, and natural and cultural exploration). They do not value the role of cruise tourism in socializing, social respect, or shopping. They are sensitive to the sources of cruise travel information and are like international cruise passengers. According to Plog’s psychological type model, this type of tourist has psychological characteristics of the mid-centric and near-allocentric type, and easily accepts cruise travel products. For this type of tourist, promotion of the product image of relaxation, exploration, and cruise travel’s multi-destination experience through a variety of information sources will likely motivate their cruise travel participation.

## Discussion

In general, the mean analysis and cluster analysis found that, on the one hand, cruise passengers in mainland China exhibited motivations consistent with the conclusions of most international cruise studies (e.g., motivation for “leisure/recreation” and for “natural and cultural exploration”) (Lu, 2001; Teye and Paris, 2010; Prebensen et al., 2013; Lemmetyinen et al., 2016; Han and Hyun, 2018), but, on the other hand, Chinese cruise passengers were found to regard cruise tourism as a traditional form of outbound travel, and uniquely exhibited motivations for “shopping” rather than enjoying a cruising experience (Sun et al., 2019a). As explained by Sun et al. (2014) and Hung et al. (2020), cruise tourism is in an early stage of development in China, the country still lacks a vibrant cruise culture, cruise promotion and publicity is insufficient, and, thus, information sources have a relatively weak influence on tourists’ motivation to pursue cruise travel. This creates travel structural constraints to cruising. Therefore, the cruise industry should first increase and accelerate the publicity and promotion of cruise products in an effort to cultivate a national cruise culture in China. Additionally, creating a desire scale for measuring Chinese cruising constraints among different market segments is a matter of urgency (Hung et al., 2020).

The study also found that other people’s word-of-mouth recommendations were the most influential information source motivating cruise passengers. The cruise market should consequently enhance customer satisfaction through the provision of high-quality tourism products and services, thereby promoting tourists’ word-of-mouth recommendations and their intentions to revisit. Repetitive purchase behaviors and positive word-of-mouth are favorable to a cruise line. According to the law of the vital few or the 80/20 rules, 20% of clients usually generate 80% of sales. In doing so, cruise lines can keep 20% of their repeat customers, and their revenue and market shares will be secured in a competitive cruise market (Sun et al., 2019a). In addition, travel agencies, cruise companies, travel websites, and social media (e.g., Instagram), have a great impact on cruise passengers, and cruise companies can utilize travel agency distribution channels and online media to increase product promotion. With the acceleration of the cruise market and the continuous expansion of the use of social media, cruise companies and their partners should increase the breadth and depth of publicity via online channels and strengthen promotion of the cruise’s core functions, such as family leisure vacation, natural and cultural exploration, escaping from daily affairs, physical and mental relaxation, and enhancing family relationships, via an abundance of information sources. Indeed, the technological transformation and changes of consumers mix and behaviors create the social transformation phenomenon in the cruise industry. In a context of social transformation, the young generation (i.e., Gen Z) becomes an emerging market segment in the cruise industry. They rely much on various online channels and innovative technologies during a cruise trip. In order to provide customized and fantastic cruising experiences, cruise lines may provide Instagramable cruise travel and onboard smart technology (e.g., key chains, apps, necklaces, and bracelets) (Lau et al., 2021).

In addition, cruise companies should adopt product promotion strategies targeting different types of tourists, so that these companies can motivate a wide range of cruise passengers effectively. Sun et al. (2018) addressed that price promotions need to align with the level of product availability in the respective behavioral segment. Indeed, cruise lines need to adopt product line pricing strategy which sets up and adjusts prices of various products within a product line according to cost differences between the products. The current study found that cruise passengers can be classified into four main types based on travel motivation: information-insensitive, complexly sensitive, complexly insensitive, and respect-insensitive types. Since the current cruise market in China is in an early stage of development, the cruise industry should first adopt a full-scale holistic approach for publicity and promotion covering all types of tourists (Lau and Yip, 2020). Such a strategy would increase awareness of cruising for Chinese tourists and promote the distinct characteristics of cruise travel, such as relaxation, entertainment, novelty seeking, vacation, sightseeing, and even shopping (Sun et al., 2014). In order to quickly introduce and stimulate the cruise tourism culture, companies should, more specifically, market personalized family leisure travel products, such as domestic game products, products of parent-child bonding activities, marine leisure life experience products, domestic DIY experience products, unique tourist souvenirs, etc., or even provide full video-capturing services for cruise travel to enhance the travel experience for tourists. To the best of our knowledge, solo, Gen Z, and female travelers have become an increasing trend in the new customer segment. Cruise lines can provide a tailor-made cruising package. Such as, arranging networking and designing far-reaching destinations for solo tourists; linking women with other women via designing cruise itineraries for women; providing different unique experience like music festivals at sea and destinations for Gen Z (Lau et al., 2021). This will stimulate travel motivation and promote positive word-of-mouth recommendations.

In order to expand the customer market in the short term, cruise companies should pay attention to the characteristics of “complexly sensitive” and “information-insensitive” tourists in their motivation for travel shopping. When selecting port destinations and developing tourism products, they should attempt to provide opportunities for duty-free shopping, as well as for purchasing souvenirs unique to each travel destination. According to Lau and Yip (2020), the authors proposed the CRUISE PORT framework to highlight the importance of connectivity between shopping areas (e.g., souvenir shops and boutiques) and cruise port areas. This will stimulate tourists’ motivation for travel shopping and thereby help the local market to extend the cruise industry chain and enhance its economic radiation effect. It should be noted that, although outbound shopping is an important factor motivating Chinese tourists to participate in cruise tourism, it should not be excessively promoted in the long run. This is not conducive to the healthy development of the cruise industry. Otherwise, it will adversely affect cruise port utilization and the home port position in the future. In addition, with the rejuvenation of cruise ship tourism worldwide, younger generations have become increasingly involved in cruise travel. Cruise industries should strengthen the product features of “cruise tourism that can fully satisfy all the leisure needs of young tourists.” In addition, the industry should also strengthen the planning of cruise products and related activities, such as current popular entertainment, variety shows, exciting challenging cruise events, or themed cruise routes. Furthermore, when appealing to cruise passengers who are “complexly insensitive” and “respect-insensitive,” companies should emphasize the characteristic features of cruise travel, such as family time, leisure, natural and cultural exploration, and especially the advantages of integrating multiple destinations.

## Conclusion

In recent years, the international cruise industry has quickly shifted from North America and Europe to the Asian Pacific. Under this context, the Chinese cruise industry has experienced an unprecedented rate of development, and the number of cruise ships and tourists has grown rapidly. At present, China’s cruise tourism industry is still in its early stage of development: the country still lacks a vibrant cruise culture, has a relatively low awareness of available cruise products, and the cruise market’s penetration rate is extremely low. Therefore, learning how to continuously attract residents of mainland China to cruise tourism is key to ensuring the sustained prosperity of the Chinese cruise industry, and developing an in-depth understanding of Chinese cruise passengers’ travel motivations is a prerequisite for developing a large source market.

For this reason, the current paper examines and categorizes various travel motivations of Chinese cruise passengers by means of a questionnaire via factor analysis, mean analysis, and K-cluster analysis. The results of the study indicated that Chinese cruise passengers are primarily encouraged to participate in cruise tourism by the motivational dimensions of family recreation/leisure, natural and cultural exploration, bond/communication, social respect, tourism shopping, and cruise-promotion information sources. The strongest motivations for Chinese cruise passengers were found to be family leisure/relaxation and natural/cultural exploration.

The study further found that other people’s word-of-mouth recommendations and “just wanting to travel” had a great impact on tourists’ engagement in cruise travel. The main implication for the cruise industry is as follows: during its initial stage of development, China’s cruise tourism industry must effectively stimulate cruise passengers’ participation and continuously expand the source market. Consequently, the cruise industry must rely on modern information networks and media technologies to better promote the product characteristics of cruise travel, such as “escaping from daily affairs,” “relaxation of the body and mind,” “enhancing family relationships,” “multi-destination experience,” and “outbound travel shopping.” In order to gain positive word-of-mouth recommendations and to encourage tourists to rebook, the industry should also focus on enhancing customer satisfaction through effective product design, event planning, and service provisions.

To the best of our knowledge, travel motivation has been widely adopted in tourism literature. However, it is very inadequately related to cruise tourism. In cruise tourism research, this is a groundbreaking study that investigates cruise passengers’ motivation in an emerging market, notably in China. We employed an emerging market as the context of our research to identify the movement in destination marketing. Analyzing the motivations of cruise passengers provides quality information for cruise lines and for destination management about how cruise passengers perceived the destination. In the long term, it can support the development of emergent cruise destinations and a port of call (Lemmetyinen et al., 2016). Theoretically, our study validated the motivation of mainland Chinese cruise passengers. To a certain extent, it can help us build a constructive research model including key variables like behavioral intentions, involvement, perceived value, and brand awareness. Further, our studies should be segmented based on demographic and socioeconomic characteristics and motivational and attitudinal variables. This study has identified four major types of cruise passengers, the “psychocentric tourist,” the “traditional tourist,” the “pioneer,” and the “sightseer” to better understand the travel motivation of different types of tourists based on Plog’s psychological type model. These findings suggest that the cruise industry should target the integrated target market of “pioneer” and “psychocentric” types of cruise passengers and employ different marketing strategies to target the other types. In addition, tour operators, marketers, and cruise lines may use the findings to motivate Chinese cruise passengers to travel. Motivation data are crucial for cruise lines, marketers, and tour operators in order to plan for the future (Jones, 2011). Indeed, our study carried out a cluster analysis concentrating on the demographic data on cruise passengers as an illustrative example of an emerging cruise destination to create their key features, which would enable the cruise lines, destination managers, and marketers to implement a targeted marketing strategy. For instance, how to allocate cruise itineraries for different segments? How to design various cruise package and delivery images of cruise products to an emerging market? How to design and implement marketing strategies for attracting more Chinese cruise passengers?

Furthermore, some limitations that should be considered in future research are present in our research. Firstly, self-reported data on tourists’ travel motivation were employed which may be subject to prejudice relevant with report accuracy and willingness to answer. Tourists may be reluctant to provide their real travel motivation because of inadequate knowledge or possible personal repercussions. Secondly, the data were mainly gathered from tourists at the Shanghai Wusongkou International Cruise Port. In the future, we may consider collecting qualitative data via focus group discussion or semi-structured face-to-face interviews to produce in-depth data analysis and obtain broader prospects. Thirdly, our study mainly focused on one Chinese city and a limited segment of Chinese tourists. Thus, the sample may not completely represent the whole of mainland China. Thus, it influenced the generalizability of the results. In order to minimize any possible bias, we may conduct a large-scale data collection in different cities in China so that we can generalize our study in the forthcoming years. Finally, we may investigate potential cruisers’ behavior on the basis of a preference model in the Chinese cruise market in a future study as we found that few studies have been undertaken in cruise tourism (Kawasaki and Lau, 2020).

## Data Availability Statement

The original contributions presented in the study are included in the article/supplementary material, further inquiries can be directed to the corresponding author.

## Ethics Statement

The studies involving human participants were reviewed and approved by College of Business Administration, Shanghai Business School. The patients/participants provided their written informed consent to participate in this study.

## Author Contributions

YJ and YH contributed to the conceptualization. YH and Y-yL contributed to the methodology. YJ and Y-yL contributed to the validation. YJ and YH contributed to the formal analysis. YJ, YH, and Y-yL contributed to the investigation. YH contributed to the data curation. YJ, YH, and Y-yL contributed to the writing—original draft preparation. YJ and Y-yL contributed to the writing—review and editing. YJ and Y-yL contributed to the project administration. All authors have read and agreed to the published version of the manuscript.

## Conflict of Interest

The authors declare that the research was conducted in the absence of any commercial or financial relationships that could be construed as a potential conflict of interest.
